# Integrating Untargeted and Targeted Metabolomics Coupled with Pathway Analysis Reveals Muscle Disorder in Osteoporosis on Orchiectomized Mice

**DOI:** 10.3390/molecules28062512

**Published:** 2023-03-09

**Authors:** Fei Ge, Ziheng Wei, Yanting Che, Qingqing Qian, Jinfei Song, Hongxia Zhao, Si Wu, Xin Dong

**Affiliations:** 1 College of Sciences, Shanghai University, Shanghai 200444, China; 2 School of Medicine, Shanghai University, Shanghai 200444, China; 3 Department of Orthopedics, Shanghai General Hospital, School of Medicine, Shanghai Jiao Tong University, Shanghai 201620, China; 4 Institute of Translational Medicine, Shanghai University, Shanghai 200444, China; 5 Department of Pharmacy, The Fifth People’s Hospital of Shanghai, Fudan University, Shanghai 201100, China; 6 National Institute of Clinical Research, The Fifth People’s Hospital of Shanghai, Fudan University, Shanghai 201100, China; 7 Zhanjiang Institute of Clinical Medicine, Central People’s Hospital of Zhanjiang, Zhanjiang 524037, China; 8 Department of Genetics, Stanford University School of Medicine, Stanford, CA 94305, USA

**Keywords:** male osteoporosis, muscle disorder, fracture prevention, testosterone, metabolomic

## Abstract

Most osteoporosis (OP) fracture accidents in men are due not only to a low BMD but also because of unhealthy muscle support. However, there has been a limited number of reports about how muscle metabolism is disturbed by OP in males. In this work, a pathway analysis based on metabolomic research was carried out to fill this gap. A classical orchiectomy procedure was adapted to create an OP animal model. A micro-CT and pathological section were applied for a bone and muscle phenotype assessment and a pathology analysis. UPLC-Q-TOF/MS and UPLC-QQQ-MS/MS were applied to measure metabolites in skeletal muscle samples among groups. In total, 31 significantly differential metabolites were detected by comparing healthy models and OP animals, and 7 representative metabolites among the 31 significantly differential metabolites were identified and validated experimentally by UPLC-QQQ-MS/MS (xanthine, L-phenylalanine, choline, hypoxanthine, L-tryptophan, succinic acid, and L-tyrosine). An ingenuity pathway analysis (IPA) analysis revealed significantly enriched pathways involved in inflammation, oxidative stress, and necrosis. To our best knowledge, this is the first study to investigate early muscle disorder processes in Cases of OP at a metabolic level, facilitating early intervention and protection from OP fractures for aged men.

## 1. Introduction

In comparison with postmenopausal osteoporosis (PMOP), OP in older men receives less attention. OP is often not diagnosed in aged men until patients exhibit hypogonadal symptoms that are associated with decreased testosterone levels [1,2]. Once OP occurs in testosterone-deficient men, it can cause significant pain and danger to the patients. Statistically, OP has an estimated prevalence of 18% in men, while 30% of osteoporotic fractures (OPF), also known as fragility fractures, occur in men [2,3,4]. This rate increases beyond 30% with aging among the population [4,5]. The main risks associated with OPF come not only from a reduction in bone mineral density (BMD) with aging but from the danger of accidental falls [6].

A low BMD puts individuals at risk of fragile fractures, which can be further aggravated by falling accidents. Dysfunctional muscles contribute significantly to the main risk factors of falls [7,8]. In a study by Yu et al., it was found that muscular dystrophy is an independent risk factor for male fractures (HR: 1.87 (95% CI: 1.30 to 2.68)) [9]. Therefore, the health condition of the muscles is crucial when developing treatment for a fragility fracture. Interestingly, muscle dysfunction is commonly observed in OP patients with low testosterone levels [10,11,12,13,14]. Severe muscle dysfunction might induce sarcopenia and myopenia. A study on 117 individuals with a high risk of OP revealed a significant correlation between skeletal muscle mass index and BMD (r = 0.46 − 0.59, *p* < 0.001) [13]. Another study on a clinical cohort with 232 older male individuals indicated that a decline in muscle strength was associated with a decrease in spine areal BMD (adjusted OR = 2.93 (1.21–7.12)) and hip areal BMD (adjusted OR = 3.42 (1.37–7.64)) [14]. Individuals with osteoporosis have an risk of complications of muscle dysfunction that is up to five times higher [14]. These close connections between the bone environment and muscle tissue are also verified in endocrinology research. Schindeler et al. identified muscle as the second periosteum layer, and muscle stem cells play an essential role in bone repair [15]. Over the past decades, the muscle–bone crosstalk theory has evolved from purely biomechanical feedback to biochemical system [9,10,16]. Muscles and bones are known to be secretory tissues that release regulative soluble molecules [17,18]. The strong positive associations between muscles and bones are evident in clinical fracture treatments. Muscle flaps have been shown to assist in the healing of open tibial fractures in mice and humans when used to cover the wound [19,20]. In addition, skeletal muscles can interact with bone through myokines in endocrine circulation via muscle contractions [21,22]. Due to a lack of attention paid to muscle health among OP patients, the sarcopenia and myopenia induced by OP have received less treatment than other complications [23]. Although studies have found that there is an early disorder of the muscles in Cases of OP, there are few works that report on the disorder from a metabolomic aspect [10,24,25,26]. Therefore, it is important to study how muscle disorder forms in cases of OP from an early stage so that proper treatments can be applied to prevent muscle dysfunction. However, only a few studies have reported the prodromal abnormality of skeletal muscles under osteoporotic conditions.

In recent years, mass spectrometry (MS) coupled with a liquid chromatogram (LC-MS) system has been widely employed by metabolomic researchers for its fast, sensitive, and wide collection capability [25,27,28,29,30]. For chronic abnormalities in the initial state, such as muscle disorders in cases of OP, metabolomic research based on the LC-MS system is one of the best methods for monitoring early unhealthy signals. There have been very few metabolomic studies that focus on muscle dysfunctions in cases of OP, especially in male OP [24,25].

Traditionally, an orchiectomy (ORX) procedure is employed to construct a testosterone-deficient animal model. It has been recognized as an OP model in rodents [28,31,32,33,34,35]. Supplementing with testosterone has demonstrated a positive effect on reversing BMD loss in testosterone-deficient individuals [36,37]. In this study, testosterone was supplemented in orchiectomized mice to compensate for the rapid decline in testosterone levels. We combined the targeted metabolomics with untargeted metabolomics to improve the accuracy of the untargeted metabolomics through the quantitative verification of key metabolites. Pathway analyses were performed to deepen our understanding. Our study utilized a combination of untargeted and targeted metabolomics to demonstrate the disturbance of muscle metabolism by OP at an early stage where severe and obvious muscle dysfunction had not yet occurred [38,39,40]. Comparing the differential metabolites in the skeletal muscles of healthy and OP models can shed light on the early pathological and physiological states of the muscle system under the conditions of OP [41,42,43].

## 2. Results

### 2.1. Assessment of OP Modeling

#### 2.1.1. Micro-Computed Tomography Assessment

**Figure 1:**
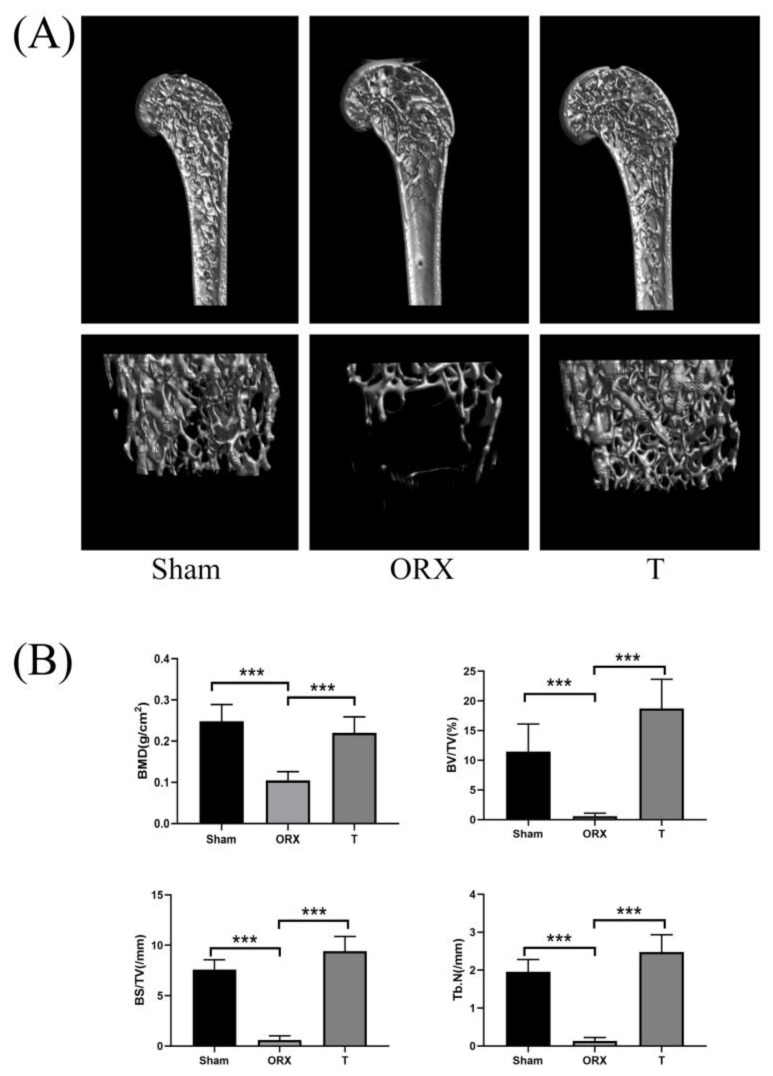
Testosterone (T) prevents bone loss in orchiectomy (ORX) mice in vivo. (**A**) Representative 3D micro-CT reconstructions of mouse femurs from sham (PBS injection), ORX (PBS injection), and T (ORX with 12.5mg/kg testosterone). (**B**) Quantitative bone morphometric parameters of bone mineral density (BMD, g/cm^2^), bone volume to total tissue volume (BV/TV), bone surface to tissue volume (BS/TV, mm^−1^), and trabecular number (Tb.N., mm^−1^) were measured. Values presented as the mean ± standard deviation (n = 3). ***: *p* < 0.001.

BMD (g/cm^2^), bone volume to total tissue volume (BV/TV), bone surface to tissue volume (BS/TV, mm^−1^), and the trabecular number (Tb.N., mm^−1^) were measured by micro-CT. Three-dimensional reconstructions of the femoral bones were obtained during the same process. The three-dimensional reconstructions showed that the ORX group demonstrated a decrease in the bone volume, trabecular bone, and all other measured, quantitative bone morphometric parameters after the ORX procedure (Figure 1A,B) when compared with the Sham group. These parameters were restored to notable extent in the T group. Therefore, the ORX procedure successfully established an OP model and the supplementation of T after the ORX procedure prevented a further drop in BMD.

#### 2.1.2. Femur Section Staining Histopathology

The HE staining of the femur revealed a higher number of adipocytes in the ORX group compared with the Sham group. The T group exhibited a reduction in adipocytes, indicating a recovery from the adipocyte overload, as shown in Figure S3A,B. An increase in adipocytes is one of the main characteristic features of OP and is indicative of an inhibited osteoblast maturation of [44]. The recovery of the number of adipocytes following testosterone treatment suggests that it is an effective intervention for symptoms of potential muscle dysfunction resulting from androgen loss.

The same trend was observed in the Trap staining, shown in Figure S3A,B. The number of TRAP+ osteoclasts and osteoclasts increased in the ORX group, while this number decreased in the T group. This finding indicates an abnormal osteoclast differentiation and greater bone resorption over bone formation. An excess number of osteoclasts is the main cause of reduced BMD and an imbalance between bone resorption and bone formation, inducing the development of OP.

### 2.2. Body, Prostate Weights, and Clinical Observation

During the experiments, the body weight of each mouse was measured and recorded for further analysis, as shown in Figure S1A. Clinical observations were carried out during the weighing process. There was a significant difference in weight between the groups at the time of sacrifice. The Sham group showed a tendency of growing, while the ORX group showed a significant decline. The T group synchronized with the Sham group. Therefore, the effect of weight loss due to the ORX procedure was obvious, and testosterone treatment could restrain this decline in weight. The healthier body weight in the Sham group and T group than the ORX group indicates a co-occurrence of unhealthy body mass index with OP.

Prostate weighing was also included before the sacrificial process, shown in Figure S1B. Statistical differences were found between the T and ORX groups after 6 weeks of T treatment. The decline in prostate weight indicates the success of the ORX procedure. The T group had the maximum prostate weight, exceeding the other two groups. By connecting these relationships, the androgen-deficient model was successfully contracted via ORX procedure. As another mean to measure muscle health status, the thigh diameter of each mouse was measured. As is shown in Figure S2, the ORX group had a significant decline in thigh diameter, indicating muscle disturbance.

A low BMI index is a sign of low defense against OP as well as of potential muscle dysfunction [45]. Taking the assessment of the OP model into consideration, it is logical to extend our work into a muscle study of androgen-deficient cases of OP.

### 2.3. Untargeted Metabolomic Profiling Analysis

The above-mentioned optimized UPLC-Q-TOF/MS method was used to obtain metabolomics data on the mouse skeletal muscles. As shown in Figure S4, the method presented total ion chromatogram (TIC) pictures, which reflect the total ions detected by UPLC-Q-TOF/MS in the Sham, ORX, and T groups. Raw data obtained from the MS results were processed using the procedures described in Section 4.6. The numbers of features obtained from the positive and negative modes were 5266 and 1225, respectively.

To evaluate the stability of the QC samples, an unsupervised PCA-X analysis was performed. Figure S5 demonstrates well the aggregation in the QC samples observed from the PCA. Therefore, it can be concluded with certainty that the LC-MS system maintained good stability throughout the analysis.

**Figure 2:**
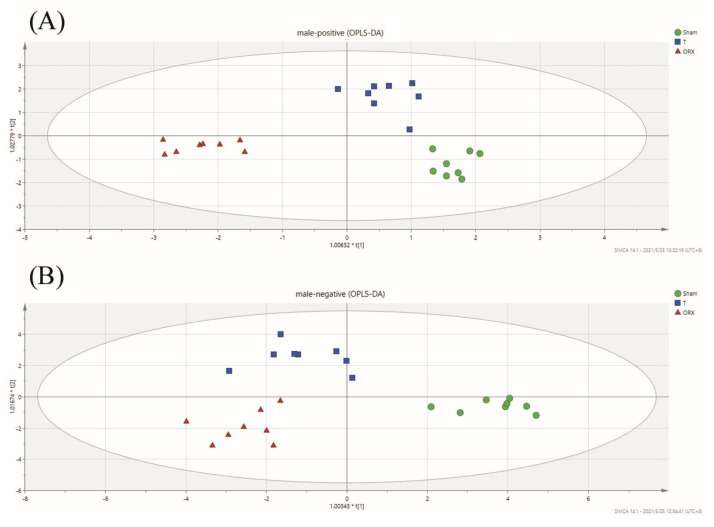
Score scatter plots for OPLS-DA analysis in (**A**) positive mode and (**B**) negative mode. The asterisk (*) in front of the horizontal and vertical axes labels of the OPLS-DA score plot indicates that the corresponding scores are scaled scores.

To determine the metabolite difference and the clustering of the Sham, ORX, and T groups, a discriminant analysis of orthogonal correct partial least squares method (OPLS-DA) analysis was employed, as shown in Figure 2 (Positive mode: R2X = 0.694, R2Y = 0.914, Q2 = 0.475; Negative mode: R2X = 0.841, R2Y = 0.886, Q2 = 0.569). The OPLS functions as a regression modeling method using multiple dependent variables and multiple independent variables [46]. It provides an interference-free environment for clustering analysis by recognizing the ions most constructive to the clustering samples. The relevant parameters R2X, R2Y, and Q2 of the OPLS-DA model were monitored to evaluate the goodness of fit and prediction internally, and the permutation tests were implemented to evaluate the quality of models externally. The expected R2 and Q2 values, which are highly dependent on their application models, should be more than 0.5 and 0.4, respectively, for a significant biological model [47]. The outcome of the OPLS-DA analysis showed that there was a tendency of separation between the groups among the Sham, ORX, and T groups and a very apparent aggregation tendency within each group. In contrast to what was observed for the Sham group, the ORX group showed some level of deviation and was shifted notably towards the T group, indicating the effects of testosterone treatment on the ORX model. Furthermore, a pairwise OPLS-DA analysis illustrated clear clustering among the groups, as depicted in Figure 2.

Figure S6E–H shows the S-plot, which displays the ions contributing to the separation of the two groups. Each ion is represented as a point on the S-plot, and the VIP value indicates the significance of the ion in the differentiation of the two groups; the greater VIP represents a larger ion differentiation between the two groups [30]. Therefore, an analysis of variance as a significant evaluation can be carried out. We screened the ions that distinguished between the Sham and ORX groups. Between the ORX and T groups, only ions of *p* < 0.05 and VIP > 1 could be retained. These differential ions were identified by applying the exact molecular weights, which were confirmed by extracted ion flow chromatographs (EIC). By comparing those exact molecular weights with common online databases, including the Human Metabolome Database (http://www.hmdb.ca/ (accessed on 7 November 2021)) and Metlin (http://metlin.scripps.edu, accessed on 7 November 2021), the identification of untargeted metabolomics was carried out as described in our previous work [46]. Differential metabolites between the Sham and ORX groups and between the ORX and T groups revealed a comprehensive understanding of changes in testosterone loss and recovery treatment.

The OPLS-DA analysis shown in Figure S6A–D reveals a significant inter-group difference and intra-group aggregation, indicating the successful establishment of the ORX process and the development of the ORX model group.

**Figure 3:**
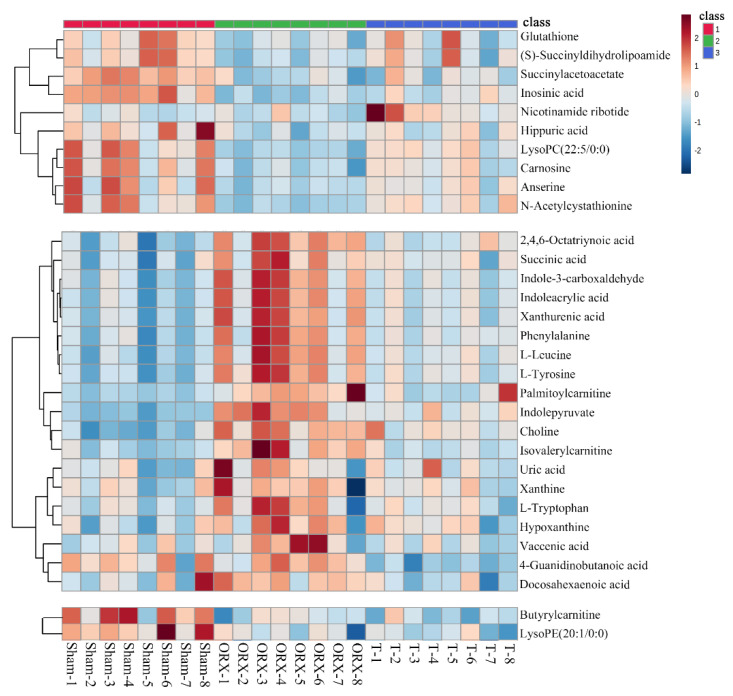
The clustering heatmap of Sham, ORX, and T group based on the 31 differential metabolites in skeletal muscle. Metabolites are divided into three groups according to the vary trend via androgen loss-and-regain process. Class1: Sham group; Class 2: ORX group; Class 3: T group.

In total, 31 significantly differential metabolites were identified during the testosterone loss-and-regain process. Their detailed information, including *m*/*z*, retention time (RT), formula, variable important in projection (VIP), fold change, related pathway, and variation, is listed in Table 1. They were divided into four groups according to their different patterns of change in the testosterone loss-and-regain process. The corresponding heatmap in Figure 3 provides a visual representation of the changing trends.

**Figure 4:**
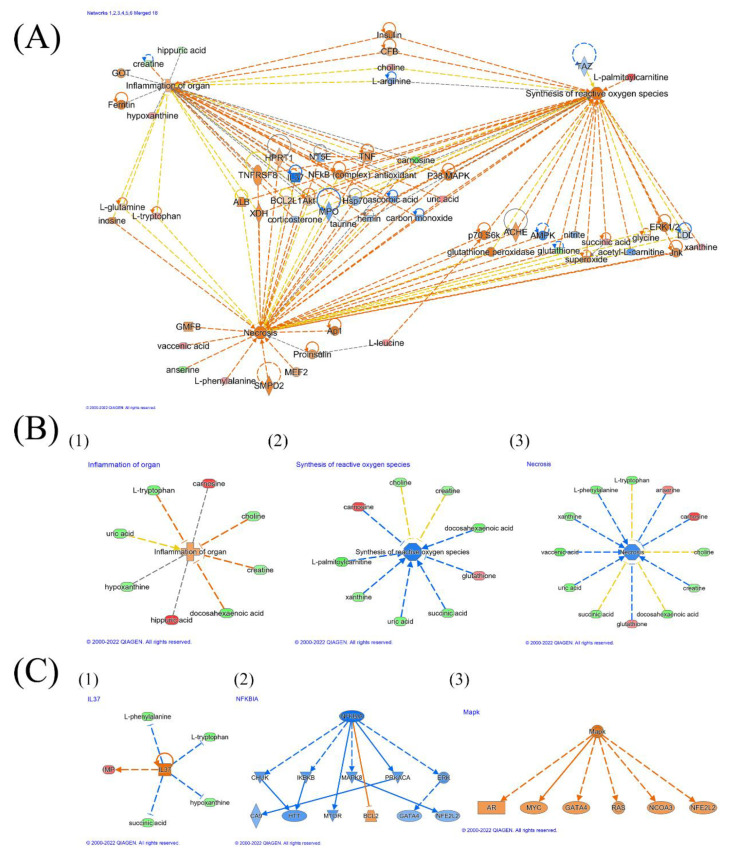
(**A**) Network analysis of called-back metabolites in this work. (**B**) Network analysis of symptoms of (1) organ inflammation, (2) synthesis of reactive oxygen species, and (3) necrosis. (**C**) Network analysis of pathological factors of (1) IL-37, (2) NFkBIA, and (3) MAPK.

A pathway enrichment analysis was conducted using Metaboanalyst 5.0 [30], as shown in Figure S7. Coupled with IPA analysis, Metaboanalyst 5.0 can provide a view to a series of inner condition variances in a testosterone-deficient mouse, as shown in Figure 4. The corresponding pathway diagram of OP from muscle–bone crosstalk was constructed as shown in Figure S8. These metabolites are closely interconnected with inflammation, oxidative stress, and necrosis. Their strong logical and biochemical interaction explains the occurrence of these three elements. The pathway analysis in Figure S8 supports the conclusion that disorders including phenylalanine metabolism, histidine metabolism, and purine metabolism are closely related to inflammation [48,49,50]. Moreover, the reduction in the levels of carnosine and anserine provide evidence of an anti-oxidative process in which oxidative stress is aggravated and the risk of necrosis is hastened. [38,39,40]. The activation of key factors such as IL37, NFkB, or Mapk provides more danger hidden in cases of OP [16,38,41,42,43]. Necrosis, in addition to these factors, is a risk for the acceleration of apoptosis in muscle cells [38,51,52]. This can, in turn, worsten the health condition of muscle cells, thereby leading to more severe inflammation. This vicious cycle increases the likelihood of myopenia and sarcopenia [53,54]. Nonetheless, these perturbated pathways were restored to some extent in the T group, indicating the beneficial effects of testosterone on muscle dysfunction in the early stages of an OP model. Our targeted validation of xanthine, L-phenylalanine, choline, hypoxanthine, L-tryptophan, succinic acid, and L-tyrosine supplement the untargeted metabolomic results, providing more accurate and results. A detailed validation is provided below.

### 2.4. Targeted Validation of Representative Metabolites

**Figure 5:**
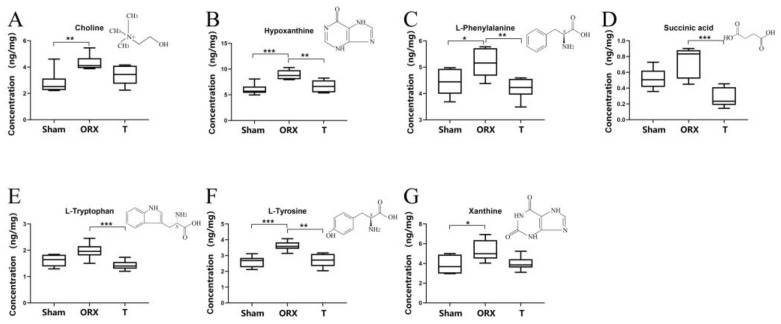
Box diagram of the concentration of different metabolites and their chemical structures (**A**–**G**) in different groups. * *p* < 0.05 vs. ORX, ** *p* < 0.01 vs. ORX, and *** *p* < 0.001 vs. ORX.

The existence of seven representative metabolites including xanthine, L-phenylalanine, choline, hypoxanthine, L-tryptophan, succinic acid, and L-tyrosine was verified by targeted multiple reaction monitoring (MRM) analysis through UPLC-QQQ-MS/MS analysis. Figure S9 shows the comparison of the retention time of these seven metabolites in the samples and standards, which confirmed the identification of these metabolites with chemical standards and aligned with the untargeted metabolomic identification. The MRM target analysis of metabolites was quantitatively determined using the respective standard solutions and the linear regression equation of peak area and concentration. The corresponding correlation coefficient, R, and low and high concentration precision correlation coefficient, R, are shown in Table 2. The absolute quantification levels of these metabolites in the three groups were consistent with the ones obtained from the untargeted metabolomic study, as shown in Figure 5. An independent sample analysis of variance was conducted to compare the concentration of each metabolite between groups after normalizing the mass of each sample. The result showed that the changes in all seven metabolites were consistent with the results obtained from the untargeted metabolomics analysis, demonstrating the reproducibility between untargeted and targeted metabolomics employed in this study.

## 3. Discussion

In this study, an androgen-deficient OP model was established in orchiectomized mice, and a rehabilitated model was constructed through testosterone treatment. Using an untargeted metabolomics analysis and targeted verification, 31 differential metabolites were identified, and seven representative metabolites were further quantitively verified in skeletal muscle samples. Furthermore, the constructed metabolic pathway network revealed a closed-loop mechanism of OP involving inflammation, oxidative stress, and necrosis in skeletal muscle. These findings suggest that OP can directly or indirectly cause alterations the levels and functions of muscle metabolic pathways. However, these disturbed pathways can be reversed in the T group, indicating the therapeutic potential of testosterone for OP.

### 3.1. Muscle Dysfunction in OP Model

The coupling of low BMD and muscle dysfunction is the main contributor to OPF [6]. In recent years, the muscle–bone crosstalk theory has been expanded from its biomechanical aspect to biochemical feedback [17,18]. The strong positive association between muscles and bones has been applied to clinical cases of bone fracture [19,20]. Certain myokines, functioning as soluble molecules, can shuttle through muscle and bone tissues via the endocrine system [18,55]. As muscle abnormalities are typically chronic, it is crucial to investigate the early stages of OP to identify how muscle disorders develop and prevent muscle dysfunction. However, the research on prodromal abnormality in skeletal muscles under osteoporotic conditions is still scarce.

### 3.2. Inflammation Environment

Differentiative metabolites related to inflammation include L-tryptophan (MS/MS verified), hypoxanthine (MS/MS verified), choline (MS/MS verified), carnosine, uric acid, and creatine, etc. These MS/MS verified metabolites suggest the activation/restriction of different factors related to the muscle inflammation environment as well as further signs of disorder, including IL-37, NF-kB, TNF, etc., as shown in Figure 5. From the inflammatory perspective, IL-37, which was found to be restrained after the ORX procedure, has demonstrated an ability to inhibit innate immunity and acquired immunity [43,52]. In a study conducted in 2021, Rosa et al. focused on the potential of IL-37 as a key indicator of rehabilitation-associated improvement in sarcopenia and as a possible therapeutic target [42]. An IPA analysis provided a tough speculation on the decline of taurine. Interestingly, taurine is an important factor that can counteract the negative effects of tumor necrosis factor α (TNF) by regulating inflammation and modulating the autophagic and apoptotic process in the myoblasts of rat skeletal muscle [40]. On the basis that the T group is normalized, the OP condition brought a vivid inflammatory environment to the skeletal muscle. Therefore, it can be concluded that the disease OP led to a loss of endocrine balance protection in the ORX group, increasing the implication risk due to the amplification of the levels of inflammation. Comparing the ORX and T groups, most of the risk factors for the inflammatory environment were reversed in the latter group. As OP symptoms did not occur in the T group, it can be concluded that inflammation is one of the early disorder mechanisms underlying muscle tissue disorders in OP.

### 3.3. Oxidative Stress

As shown in the pathway enrichment results presented in Figure S8, the histidine metabolism level in skeletal muscles appeared to drop significantly in the ORX group of the OP mouse model, whereas the levels of anserine and carnosine showed a strong elevation. Dietary studies suggest that the intake of anserine and carnosine benefits diseases related to aging, including bone health issues. Previous research demonstrated that anserine is the methylated product of carnosine, and the anti-oxidative activities of anserine and carnosine has been well proven [56]. Evidence also suggests that anserine, rather than carnosine, inhibits the activity of carnosinase, offering a better performance for carnosine [57]. In this study, the observed drop in skeletal muscle carnosine and anserine levels indicates a gap in the anti-oxidative process during the OP disease. In the OP-freed T group, the reverse of this trend demonstrates a flagrant improvement. Notably, testosterone treatment can stimulate carnosine synthesis in skeletal muscles [58]. Moreover, the decline and reversal in lysophospholipids (LysoPC/LysoPE) indicates that the lack of androgen caused a disordered skeletal muscle glycerophospholipid metabolism. LysoPC can travel from skeletal muscle cells to osteocytes via multiple paths, resulting in the generation of reactive oxygen species and leading to oxidative stress [29,59]. Once oxidative stress is activated, the muscle dysfunction will accelerate [3,20]. Additionally, OP has caused significant purine metabolism disorder, particularly with variations in xanthine (MS/MS verified), hypoxanthine (MS/MS verified), urate, inosine, and inosine acid. Except for a dropping trend observed in the level of inosinic acid, the level of muscle purine metabolism in the ORX group rose when OP symptoms were detected. Among all the differential metabolites in this study, xanthine and hypoxanthine are typical oxidative stressors [60]. Considering that hypoxanthine is derived from inosine metabolized by purine nucleoside phosphorylase activity [61], the growth of inosine, xanthine, and hypoxanthine all represent signs of oxidative stress in the muscle tissues of OP mice. Based on a study on the association between BMD and metabolome of the Chinese population in 2020 [36], high blood levels of inosine and xanthine are related to a low BMD. After intervention with testosterone, this trend is reversed, and purine metabolism is corrected close to the normal level. Coupled with this study, obvious oxidative stress proofs are indicators of the risk influence of OP on muscle metabolism.

A strong oxidative stress was observed from the IPA analysis shown in Figure 5. All three members of the mitogen-activated protein kinase (MAPK), ERK1/2, JNK, and P38, are closely related to the synthesis of reactive oxygen species (ROS) [62]. The activation of JNK and P38 can induce pathological cell death, and ERK1/2 was activated in response to ROS [63]. The activation of ERK1/2 will cause the ROS concentration to grow [38]. Excessive ROS levels will lead to oxidative stress in muscles, inducing a muscle–bone oxidative stress environment. Coupled with an inflammatory environment, this will accelerate the abnormality of muscle function and expose patients to a high OPF risk. These metabolic oxidative disorders were observed in OP models but not in the T group with normalized BMD, representing early symptoms of muscle dysfunction. Ignoring these metabolic disorders can be a risk sign for falling or worse outcomes.

### 3.4. TNF, TNF Receptor, and Necrosis

Combining the discussion above, the ORX procedure caused both inflammatory and oxidative stress conditions which were closely attached to the development of further disease conditions. Notably, there was a relatively complete pathway for the TNF to arise, which has been proven to increase muscle wasting [40]. This pathway is closely linked to oxidative stress and an inflammatory environment. A discussion about necrosis would bring a thorough understanding of the effect androgen deficiency has on muscle–bone crosstalk. Under normal conditions, NF-kB binds with the IkB protein in cytoplasm. Once stimulation, such as inflammatory cytokines, occurs, the release from IkB allows the NF-kB to travel into nucleus and activate many DNA expressions to realize different cellular responses [41]. TNF, as a stimulation of NF-kB, can activate NF-kB in muscle cells. This activation is closely attached to muscle cell necrosis and can induce muscle loss and a metabolic disorder [41]. Skeletal muscle diseases, including muscular dystrophy [64] and Duchenne muscular dystrophy [65], which are associated with muscle loss, are always coupled with activation of NF-kB [41]. These research studies indicated the activation of NF-kB as a common phenomenon in muscle diseases and a potential cause of muscle loss. The activation of NF-kB in the skeletal muscle of the ORX group in our study was a strong warning sign of muscle loss. It is known that NF-kB controls the formation of osteoclasts and the response of bone resorption to various stimulations [51]. Not only will the activation of NF-kB in bones influence normal muscle metabolism and increase the risk for muscle diseases, but TNF can also travel between the muscle and bone environments. In this way, OP forms a vicious circle under circumstances of androgen deficiency. Once OP and muscle dysfunction co-occur, the possibility of OPF to occur greatly increases.

### 3.5. Comparison of Muscle Disorder in OP Male Mice and in OP Female Mice

According to our former study [30], the pathway analysis results in OP male mice are commonly consistent with those in OP female mice. Purine and histidine metabolic pathways were downregulated in the OP model and called back in the sex-hormone-treated model.

However, there were significant sex differences in the lipid metabolism pathways. In male mice with osteoporosis, the level of lysoPC decreased significantly and increased with the supplementation of testosterone, indicating changes in lipid metabolism. In contrast, the regulation of the lipid metabolism pathway in female mice showed the opposite trend.

These results suggest that sex-specific differences in hormonal regulation play a significant role in the metabolic pathways associated with muscle disorders and osteoporosis. The differences in the lipid metabolism pathway between male and female mice could be attributed to the differential effects of sex hormones on this pathway. Further studies are necessary to elucidate the underlying mechanisms and implications of these sex-specific metabolic changes.

## 4. Materials and Methods

### 4.1. Chemicals and Reagents

Methanol (HPLC grade) and acetonitrile (HPLC grade) were purchased from Merck (Darmstadt, Germany). Formic acid (HPLC grade), 2-chloro-L-phenylalanine (as an internal standard), xanthine, L-phenylalanine, D9-phenylalanine, choline, hypoxanthine, L-tryptophan, succinic acid, and L-tyrosine standards were purchased from Sigma (Saint Louis, MO, USA). A Milli-Q system (Millipore, Bedford, MA, USA) was applied to produce ultrapure water.

The animal treatment used corn oil (GLBIO, Montclair, CA, USA) and testosterone (GLBIO, Montclair, CA, USA). An 80% paraformaldehyde stationary liquid (Labgic Technology, Beijing, China) was applied for sample preservation.

For the decalcification of bone tissue, EDTA-2Na and sodium hydroxide were purchased from Sinopharm Chemical Reagent Co., Ltd., Shanghai, China., We applied the same pretreatment process for Hematoxylin-eosin (HE) and tartrate-resistant acid phosphatase (TRAP) staining. Anhydrous ethanol was purchased from Sinopharm Chemical Reagent Co., Ltd. (Shanghai, China). An environmentally friendly dewaxing agent (alkane mixture), a transparent agent (alkane mixture, xylene-free), and other dye tools were purchased from Tonsen tech, Wuhan, China. For further staining, the HE dye kit was purchased from Biossci (Beijing, China), model BP092. The TRAP dye kit was purchased from Biossci (Beijing, China), model BP088.

### 4.2. Animals & Treatment

Twenty-four nine-week-old, wild-type male C57/LB6 mice were purchased from Shanghai SLAC laboratory Animal Co. Ltd. After one week of adaptive housing with a controlled temperature (22–24 °C), humidity (50–60%), a 12 h light/dark cycle, and free water and food, the mice were randomly and evenly divided into Sham, orchiectomy (ORX), and testosterone-treated (T) groups.

The mice in the ORX and T groups were put under anesthesia. All surgeries were performed in a pathogen-free animal laboratory. The ORX group and T group received an orchiectomy procedure, and the Sham group received a sham procedure in which open and suture surgeries were performed exactly where the ORX surgery would be performed on but included no resection. All the mice were given one week of recovery. From the second week, the T group began to receive testosterone injections at a dose of 12.5 mg/kg with a frequency of every 48 h. The injections were performed in the form of a cervical subcutaneous injection alternated with a quadriceps femoris intramuscular injection. The housing process was carried out for six weeks. At the end of the sixth week, all the mice were sacrificed. Upper limb (together with skeletal muscles) and lower limb samples were collected. The upper limbs were stored under −80 °C before pre-treatment. The lower limbs were immersed in 80% paraformaldehyde stationary liquid for 24 h and then changed to 75% ethanol for long-term preservation. All animals were housed in a temperature-controlled environment of 22–25 °C with a 12 h light/dark cycle and were fed standard rodent chow and water ad libitum. All animal experiments and models were approved and performed in accordance with the guidelines of the Ethics Committee of Shanghai University and the ethics approval No. is ECSHU 2021-196.

### 4.3. Micro-Computed Tomography and Histopathology Assessment

Considering the abundance of trabeculae in the distal femur, the three-dimensional analysis of distal femur was adapted for the comparison of the morphology characteristics in different groups. For micro-computed tomography (micro-CT) assessment, a Skyscan 1275 high-resolution micro-CT scanner (Bruker, Billerica, MA, USA) was adapted for three-dimensional reconstructions of the femur peeled off from the femurs. The image acquisition voltage was 50 kV, and the current was 60 μA. The isotropic resolution was 18mm. An aluminum filter with a thickness of 0.5–0.75 mm was adapted for beam-hardening reduction. The final bone parameters obtained from micro-CT assessment included bone mineral density (BMD, g/cm^2^), bone volume/total volume (BV/TV), bone surface to total volume ratio (BS/TV, mm^−1^), and the trabecular number (Tb.N, mm^−1^).

After micro-CT analysis, the fixed bone samples were decalcified in 10% EDTA for 2 weeks. They were then embedded in paraffin blocks for sectioning into 5 mm thick sections. The bone sections went through HE and TRAP staining for pathology analysis, as mentioned in our previous work (26). A light microscope (Nikon, Tokyo, Japan) was applied to image staining sections at 5× and 20× magnification.

### 4.4. Untargeted Metabolomic Analysis

Skeletal muscles from the upper limb were attained with scissors and accurately weighed (20 ± 2 mg). All the samples were mixed with 500 μL of methanol containing 5 μg/mL 2-Chloro-L-phenylalanine as an internal standard. The mixtures were placed in a high-throughput tissue grinder (Tissuelyser-24, Jingxin, Shanghai, China) in 60 Hz for 90 s, thus realizing homogenate and protein precipitation. The following step comprised the centrifugation of samples at 12,000 rpm and 4 °C for 15 min. A total of 100 μL of supernatant from each sample was obtained for further LC-MS analysis. Finally, 10 μL aliquots of each sample mixture functioned as a pooled quality control (QC).

An Agilent 1290 Infinity LC system coupled with an Agilent 6545 accurate mass quadrupole time-of-flight (Q-TOF) mass spectrometer (Agilent, Lexington, MA, USA) was adapted for UHPLC-Q-TOF/MS analysis. Chromatographic column was Waters ACQUITY UPLC HSS T3 analytical column (2.1 mm × 100 mm, 1.8 μm, Waters, Milford, MA, USA). The column temperature was 40 °C and the sample chamber temperature was set at 4 °C. The mobile phase in this experiment was acetonitrile with 0.1% formic acid (eluent A) and water with 5% ACN and 0.1% formic acid (eluent B) at 0.4 L/min. The injection volume was 5 μL. Instead of analyzing the samples in group order, all the samples were injected randomly and QC samples were analyzed once every eight injections to ensure system stability. In this case, the mobile phase consisted of the following gradient: 0–2 min: 100% (B); 2–5 min: 100–50% (B); 5–13 min: 50–15% (B); 13–14 min: 15–5% (B); and 14–15 min: 5% (B). Mass spectrometric conditions included both positive and negative ion modes. Other key parameters were set as follows: the mass range was set between 100 and1100 *m*/*z*; the fragmentor was set at 120 V; the gas temperature was at 350 °C; the flow rate of drying gas was 11 L/min; the nebulizer was set at 45 psig, and the V Capillary was 3.5 kV in positive ion mode and 3.2 kV in negative ion mode.

### 4.5. Targeted Metabolomic Analysis

The extraction of skeletal muscles was consistent with the untargeted metabolomic analysis process. After extraction, 100ng/mL of D9-phenylalanine was assured to exist in each sample as an isotopic internal standard. Standard curves of each standard were generated with a dilution of 50% ACN.

Agilent 1260 Infinity LC system coupled with Agilent 6460 Triple Quadruple (QQQ) MS/MS spectrometer (Agilent, USA) was adapted for UPLC-QQQ-MS/MS analysis. The chromatographic column was Waters XBridge BEH Amide analytical column (2.1 mm × 100 mm, 2.5 μm, Waters, Milford, Massachusetts). The column temperature was 30 ℃ and the sample chamber temperature was set at 4 °C. The mobile phase in this experiment comprised acetonitrile (0.1% formic acid) (eluent C) and water (0.1% formic acid) (eluent D) at 0.35 L/min. The injection volume was 1 μL. Instead of analyzing the samples in group order, all the samples were injected randomly, and QC samples were analyzed once every eight injections to assure system stability. In this case, the mobile phase consisted of (C) water and (D) acetonitrile in the following gradient: 0–2 min: 80% (D); 2–6 min: 80–55% (D); 6–8 min: 55–55% (D); and post time: 5 min. Scan segments are described as shown in Table 2. Other key parameters were set as follows: the gas temperature was at 350 °C; the flow rate of drying gas was 10 L/min; the nebulizer was set at 40 psig, and the capillary was set at 4.0 kV in positive ion mode and 3.5 kV in negative ion mode.

The evaluation of our quantitative method followed our previous published work [47]. Both the linear R value, accuracy, and precision were measured to evaluate the reliability of our quantitative method. In brief, the calculation of those factors followed the flow below:

(1) $$\mathrm{Linear}\;R=\sqrt{\frac{\sum^{\;}{\left(y_{j}-y_{average}\right)}^{2}}{\sum^{\;}{\left(y_{i}-y_{average}\right)}^{2}}}$$

where *y_j_* indicates the regression estimation for the peak intensity of each metabolite; *y_i_* indicates the actual peak intensity of each metabolite; and *y_average_* indicates the average value of peak intensity of each metabolite. Other indexes followed the Guidelines for Validation of Quantitative Analysis Methods for Biological Samples in Chinese Pharmacopoeia 2020 Edition.

### 4.6. Data Processing & Statistical Analysis

The raw data (D) attained from UPLC-Q-TOF/MS were transferred into a general data format (.mz data) using Agilent MassHunter workstation, software version B.01.04 (Agilent, Lexington, MA, USA). The elimination of noise was realized by setting the intensity threshold to 300, and the isotope interference was eliminated. Then, an XCMS from http://metlin.scripts.edu/download/ (accessed on 7 November 2021) was used to extract and compare the peak areas as well as generate a visualized data matrix by processing the polarity of each data. In the next step, only the ions detected in over 80% of the samples were selected for further study. The number of features in the positive and negative modes was 2769 and 695, respectively. The peak area of ions in each group was divided by the peak area of the internal standard and then divided by sample weight recorded before to realize normalization. At last, three-dimensional data matrices of three groups were submitted to a SIMCA-P program (version 11.0, Umetrics, Umea, Sweden) with their sample name, retention time, *m*/*z* pair, and normalized ionic intensities for a multivariate statistical analysis including a principal component analysis (PCA) and a partial least squares discriminant analysis (OPLS-DA). The VIP (variable importance) value, generated in PLS-DA processing, represents the contribution to the group discrimination of each metabolite. In our study, VIP values of all potential biomarkers should be greater than 1. All data were expressed by means ± SD.

The statistical significance in of the mean values was tested using one-way ANOVA through the SPSS 17.0 program (IBM, New York, NY, USA). Differences were considered significant when the *p* values were less than 0.05. A heatmap analysis and network analysis were performed using MetaboAnalyst 5.0 platform (http://www.metaboanalyst.ca (accessed on 11 November 2021).

### 4.7. Bioinformatics Analysis of Metabolites

A metabolomics profile of the differential called-back metabolites adapted QIAGEN’s ingenuity pathway analysis (IPA, QIAGEN Redwood City, CA, USA, http://www.qiagen.com/ingenuity, accessed on 2 December 2021) to investigate the corrected pathological processes in this work. An IPA analysis evaluates the results mainly by two parameters, the *p* value and the z-score. Based on the right-tailed Fisher’s exact test algorithm, the *p* value shows the association between a set of meaningful molecules in an experiment, and the known process comes from random matching, taking neither the effects of the molecules nor the fold change value between the molecules in the data set. The Z-score evaluates the effect of molecular changes during biological processes. Molecules with a z-score >2 are significantly activated and molecules with a z-score of <−2 were significantly inhibited.

## 5. Conclusions

In this study, models of androgen-deficient OP and T-treated OP were established successfully. Untargeted metabolomics are widely applied in the metabolomics field; however, untargeted metabolics usually brings difficulties to providing accurate metabolite identification with a high confidence, further yielding precise biological insights. To overcome this limitation, we combined the targeted metabolomics with untargeted metabolomics profiling for the skeletal muscles to provide more accurate metabolite identification and quantification than the untargeted metabolomics alone. In our study, we identified 31 differential metabolites in total by comparing the untargeted metabolomic data of the ORX, Sham, and T groups. The pathway analysis indicated that OP probably interrupts skeletal muscles via inflammatory reactions, oxidative stress, and necrosis. Coupled with the pathway abnormality, these three elements indicated that the absence of testosterone has always been a huge risk to induce a worsened muscle disorder. Our testosterone supplement treatment alleviated the disturbance to some extent, helping to achieve a healthy BMD and helping to restore the healthy muscle metabolism. This study aims to attract attention to early prevention and intervention of muscle metabolic disorder in aged men with OP.

## Figures and Tables

**Table 1:** Differential metabolites related to muscle–bone crosstalk and therapeutical effects of testosterone throughout the androgen loss-and-regain process.

	*m*/*z*	RT	Name	Formula	Ion	VIP		Fold Change		Pathway	Variation in Testosterone Loss-and-Regain Process
						[CON-ORX]	[ORX—T]	[CON/ORX]	[ORX/T]		
1	308.0970	0.71	(S)-Succinyldihydrolipoamide△	C_12_H_21_NO_4_S_2_	[M+H]^+^	2.13	1.27	0.52 **	1.47	-	↓-↑
2	239.1164	0.59	Anserine△	C_10_H_16_N_4_O_3_	[M−H]^−^	3.73	2.82	0.51 ***	1.42	Histidine metabolism	↓-↑
3	225.1004	0.58	Carnosine△	C_9_H_14_N_4_O_3_	[M−H]^−^	3.62	3.39	0.37 ***	2.09 *	Histidine metabolism	↓-↑
4	306.0735	0.99	Glutathione△	C_10_H_17_N_3_O_6_S	[M−H]^−^	1.44	1.19	0.62 *	1.39	Cysteine and methionine metabolism	↓-↑
5	178.0512	4.24	Hippuric acid△	C_9_H_9_NO_3_	[M−H]^−^	0.17	1.05	0.97	2.28 *	Phenylalanine metabolism	↓-↑
6	349.0559	1.00	Inosinic acid△	C_10_H_13_N_4_O_8_P	[M+H]^+^	3.49	1.69	0.30 ***	1.78 *	Purine metabolism	↓-↑
7	571.3491	8.63	LysoPC (22:5/0:0) △	C_30_H_52_NO_7_P	[M+H]^+^	1.33	0.59	0.63 *	1.15	Glycerophospholipid metabolism	↓-↑
8	265.0829	0.60	N-Acetylcystathionine△	C_9_H_16_N_2_O_5_S	[M+H]^+^	1.52	1.04	0.42 **	1.57	-	↓-↑
9	198.0863	0.57	N-Acetylhistidine△	C_8_H_11_N_3_O_3_	[M+H]^+^	1.45	1.19	0.53 ***	1.51 *	-	↓-↑
10	201.0392	1.03	Succinylacetoacetate△	C_8_H_10_O_6_	[M−H]^+^	2.92	0.83	0.77 **	1.12	-	↓-↑
11	133.0288	1.01	2,4,6-Octatriynoic acid△	C_8_H_4_O_2_	[M+H]^+^	1.66	1.50	1.44 ***	0.79 **	-	↑-↓
12	104.1085	0.63	Choline▲	C_5_H_14_NO	[M+H]^+^	5.54	3.56	1.30 ***	0.89 *	Glycine, serine, and threonine metabolism	↑-↓
13	163.1183	0.65	4-Guanidinobutanoic acid△	C_5_H_11_N_3_O_2_	[M+H]^+^	0.14	1.10	1.01	0.76 ***	Arginine and proline metabolism	↑-↓
14	329.2441	13.06	Docosahexaenoic acid△	C_22_H_32_O_2_	[M+H]^+^	0.99	1.89	1.15	0.73 *	Biosynthesis of unsaturated fatty acids	↑-↓
15	135.0315	1.01	Hypoxanthine▲	C_5_H_4_N_4_O	[M−H]^−^	2.85	2.40	1.31 *	0.89	Purine metabolism	↑-↓
16	146.0593	3.76	Indole-3-carboxaldehyde△	C_9_H_7_NO	[M+H]^+^	1.19	1.19	1.45 ***	0.74 **	Purine metabolism	↑-↓
17	188.0697	3.76	Indoleacrylic acid△	C_11_H_9_NO_2_	[M+Na]^+^	3.46	3.43	1.48 ***	0.73 ***	-	↑-↓
18	238.0265	1.09	Indolepyruvate△	C_11_H_9_NO_3_	[M+Cl]^−^	0.67	0.55	1.64 ***	0.86	Tryptophan metabolism	↑-↓
19	246.1686	4.08	Isovalerylcarnitine△	C_12_H_23_NO_4_	[M+H]^+^	3.17	2.90	2.49 ***	0.49 ***	-	↑-↓
20	132.1007	1.21	L-Leucine△	C_6_H_13_NO_2_	[M+H]^+^	13.91	12.68	1.55 ***	0.75 **	Valine, leucine, and isoleucine degradation	↑-↓
	130.0876				[M−H]^−^	2.40	5.04	0.62 ***	1.32 **		
21	203.0838	3.80	L-Tryptophan▲	C_11_H_12_N_2_O_2_	[M−H]^−^	1.52	1.42	1.48 ***	0.74 **	Glycine, serine, and threonine metabolism	↑-↓
22	182.0772	1.06	L-Tyrosine▲	C_9_H_11_NO_3_	[M−H]^−^	1.74	1.46	4.62 ***	0.81	Phenylalanine metabolism	↑-↓
23	401.3446	9.13	Palmitoylcarnitine△	C_27_H_44_O_2_	[M+H]^+^	2.76	2.27	2.73 **	0.60	Fatty acid degradation	↑-↓
24	166.0851	2.01	Phenylalanine▲	C_9_H_11_NO_2_	[M+H]^+^	8.48	8.30	1.45 ***	0.76 **	Phenylalanine metabolism	↑-↓
25	119.0328	1.02	Succinic acid▲	C_4_H_6_O_4_	[M+H]^+^	1.18	1.08	1.36 **	0.81 *	Citrate cycle (TCA cycle)	↑-↓
26	167.0199	0.85	Uric acid△	C_5_H_4_N_4_O_3_	[M−H]^−^	2.19	1.71	1.55 *	0.82	Purine metabolism	↑-↓
27	281.2512	14.64	Vaccenic acid△	C_18_H_34_O_2_	[M−H]^−^	0.89	1.07	1.56 *	0.64 *	-	↑-↓
28	151.0247	0.72	Xanthine▲	C_5_H_4_N_4_O_2_	[M−H]^−^	2.08	2.12	1.28 *	0.90	Purine metabolism	↑-↓
29	206.0993	3.76	Xanthurenic acid△	C_10_H_7_NO_4_	[M+H]^+^	1.31	1.32	1.47 ***	0.73 **	Tryptophan metabolism	↑-↓
30	232.1530	3.16	Butyrylcarnitine△	C_11_H_21_NO_4_	[M+H]^+^	1.08	0.29	0.67 **	0.92	-	↓-↓
31	506.3314	9.48	LysoPE (20:1/0:0) △	C_25_H_50_NO_7_P	[M−H]^−^	0.75	0.34	0.68 **	0.91	-	↓-↓

△: metabolites putatively annotated. ▲: metabolites validated with standards. *: 0.05 > *p* > 0.01, **: 0.01 > *p* > 0.001, ***: 0.001 > *p*, -: no significant difference between groups. For the “variation in testosterone loss-and-regain”: ↓-↑: down-and-up regulated components; ↑-↓: up-and-down regulated components; ↓-↓: down-and-down regulated components.

**Table 2:** MRM target metabolomics verification for seven metabolists.

ID	RT (min)	Compound	FC		*p* Value		Ionization Mode	PRE	PRO	CE (eV)	Fragmentor (eV)	Liner R	Accuracy	Precision (LC)	Precision (HC)
			(ORX/Sham)	(T/ORX)	(ORX/Sham)	(T/ORX)									
HMDB0000292	3.79	Xanthine	1.37	0.75	0.0372	0.0603	positive	153.0	110.0	18	85	0.9997	102.51%	0.63%	0.46%
HMDB0000159	4.45	L-Phenylalanine	1.17	0.81	0.0461	0.0092	positive	166.1	105.1	9	70	0.9999	102.51%	1.1%	0.63%
HMDB0000097	4.17	Choline	1.55	0.78	0.0086	0.1128	positive	105.1	61.2	18	80	0.9996	102.51%	0.63%	0.46%
HMDB0000157	3.56	Hypoxanthine	1.48	0.75	0.0008	0.0069	positive	137.0	55.3	34	120	0.9983	100.57%	4.1%	1.5%
HMDB0000929	4.46	L-Tryptophan	1.22	0.72	0.0508	0.0038	positive	205.1	187.9	5	65	0.9991	99.16%	0.69%	4.2%
HMDB0000254	1.50	Succinic acid	1.42	0.37	0.0585	0.0003	negative	116.7	73.0	9	70	0.9994	106.29%	7.8%	3.5%
HMDB0000158	4.93	L-Tyrosine	1.38	0.75	0.0007	0.0015	positive	182.1	136.0	10	65	0.9996	105.57%	1.5%	0.68%

FC: fold change; PRE: precursor; PRO: product ion; CE: collision energy; LC: low concentration; and HC: high concentration.

## Data Availability

Data are available upon request due to restrictions, e.g., privacy or ethical.

## Supplementary Materials

The following supporting information can be downloaded at: https://www.mdpi.com/article/10.3390/molecules28062512/s1, Figure S1: (A) Line chart of mouse body weight over time. Significant differences were observed. (B) Histogram of mouse prostate weight. * *p* < 0.05 vs. ORX, ** *p* < 0.01 vs. ORX, *** *p* < 0.001 vs. ORX; Figure S2: Thigh diameter of mice in Sham, ORX, and T groups was measured (n = 6). * *p* < 0.05 vs. ORX, ** *p* < 0.01 vs. ORX, *** *p* < 0.001; Figure S3: (A) Representative photomicrographs of femur sections stained for H&E and TRAP activity (20× magnification). (B) Quantitative assessment of the total number of adipocytes and TRAP+ cells per bone surface were conducted. Values presented as the mean ± standard deviation (n = 3); Figure S4: Representative total ion chromatograms of UPLC-Q-TOF/MS. (A) TIC of Sham group in positive mode, (B) TIC of Sham group in negative mode, (C) TIC of ORX group in positive mode, (D) TIC of ORX group in negative mode, (E) TIC of T group in positive mode, and (F) TIC of T group in negative mode; Figure S5: Score scatter plots for PCA-X analysis in (A) negative mode and (B) positive mode; Figure S6: OPLS-DA score plots of Sham, ORX, and T groups (A–D) and their corresponding S-plots (E–H). Promising degree of fitting and predictive ability make it reliable to screen the differential variables between groups: (A,E) ORX vs. Sham in positive mode; (B,F) ORX vs. Sham in negative mode; (C,G) ORX vs. T in positive mode; and (D,H) ORX vs. T in negative mode; Figure S7: Pathway enrichment results of the differentiated metabolites (A) phenylalanine, tyrosine, and tryptophan biosynthesis; (B) phenylalanine metabolism; (C) purine metabolism; (D) histidine metabolism; (E) and glutathione metabolism; Figure S8: Metabolic pathways related to the differential metabolites identified in the Sham, ORX, and T groups; Figure S9: Extracted ion chromatogram (EIC) from seven metabolites in MRM analysis. A-G: EIC of choline, hypoxanthine, L-phenylalanine, succinic acid, L-tryptophan, L-tyrosine, xanthine in standard, Sham, ORX, and T groups separately. The lab animals’ experimental approval was also posted in Supplementary Materials.
