# Supplementary material for: Integrating Untargeted and Targeted Metabolomics Coupled with Pathway Analysis Reveals Muscle Disorder in Osteoporosis on Orchiectomized Mice
Source: Molecules. 2023 Mar 9;28(6):2512. doi: 10.3390/molecules28062512 (PMC10051496; doi:10.3390/molecules28062512)
Supplement: Supplementary file 1 [file molecules-28-02512-s001.zip › molecules-2175188-supplementary.pdf]

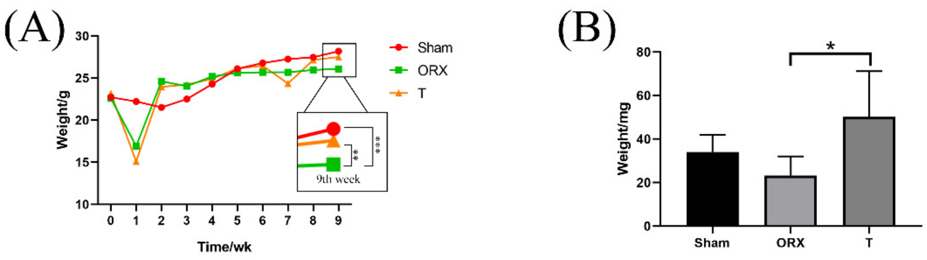

Figure S1. (A) Line chart of mouse body weight over time (B) Histogram of mouse prostate weight. *p < 0.05 vs ORX, **p < 0.01 vs ORX, ***p < 0.001 vs ORX.

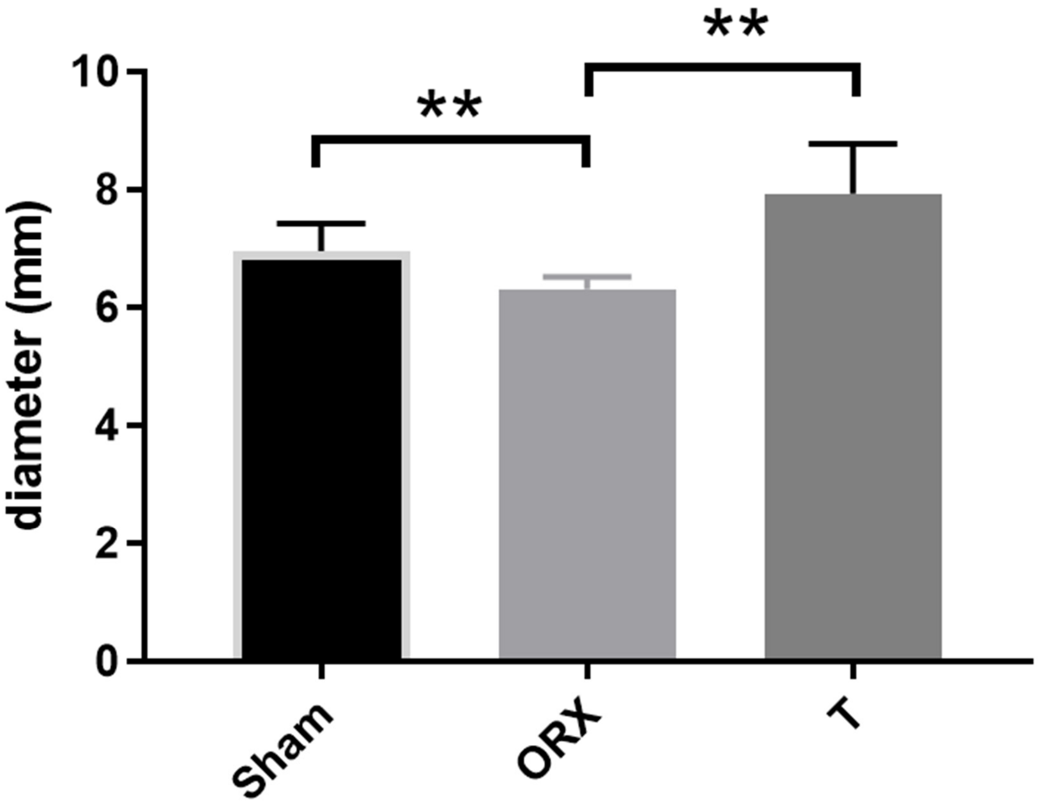

Figure S2. Thigh diameter of mice in Sham, ORX and T group was measured (n = 6).

*p < 0.05 vs ORX, **p < 0.01 vs ORX, ***p < 0.00

(A)

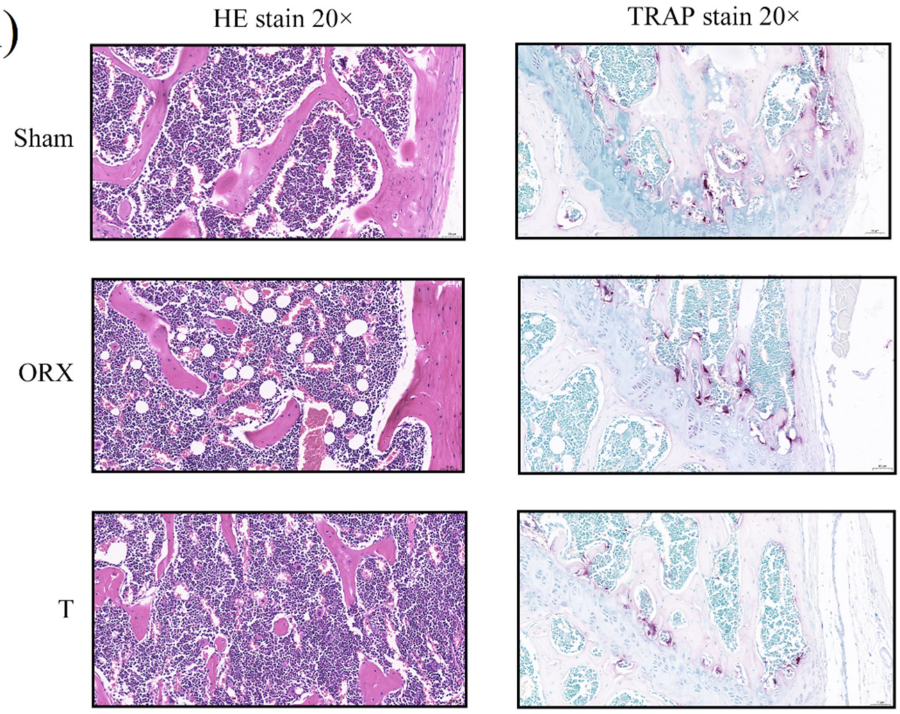

(B)

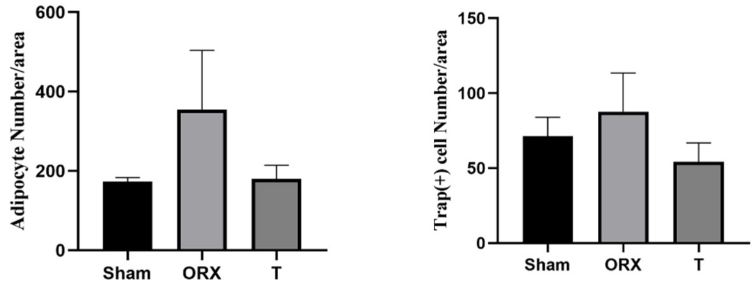

Figure S3. (A) Representative histological assessment of mouse femur sections stained for H&E and TRAP activity (20× magnification). (B) Quantitative assessment of the total number of adipocytes and TRAP+ cells per bone surface were conducted. Values presented as the mean ± standard deviation (n = 3).

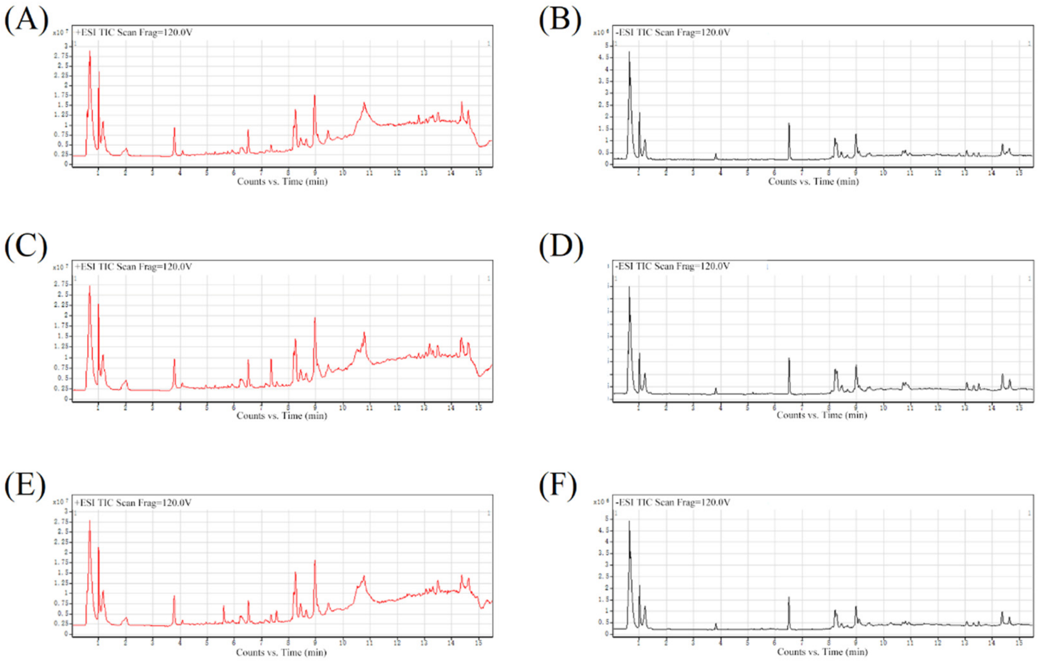

Figure S4. Representative total ion chromatograms of UPLC-Q-TOF/MS (A) TIC of Sham group in positive mode (B) TIC of Sham group in negative mode (C) TIC of ORX group in positive mode (D) TIC of ORX group in negative mode (E) TIC of T group in positive mode (F) TIC of T group in negative mode.

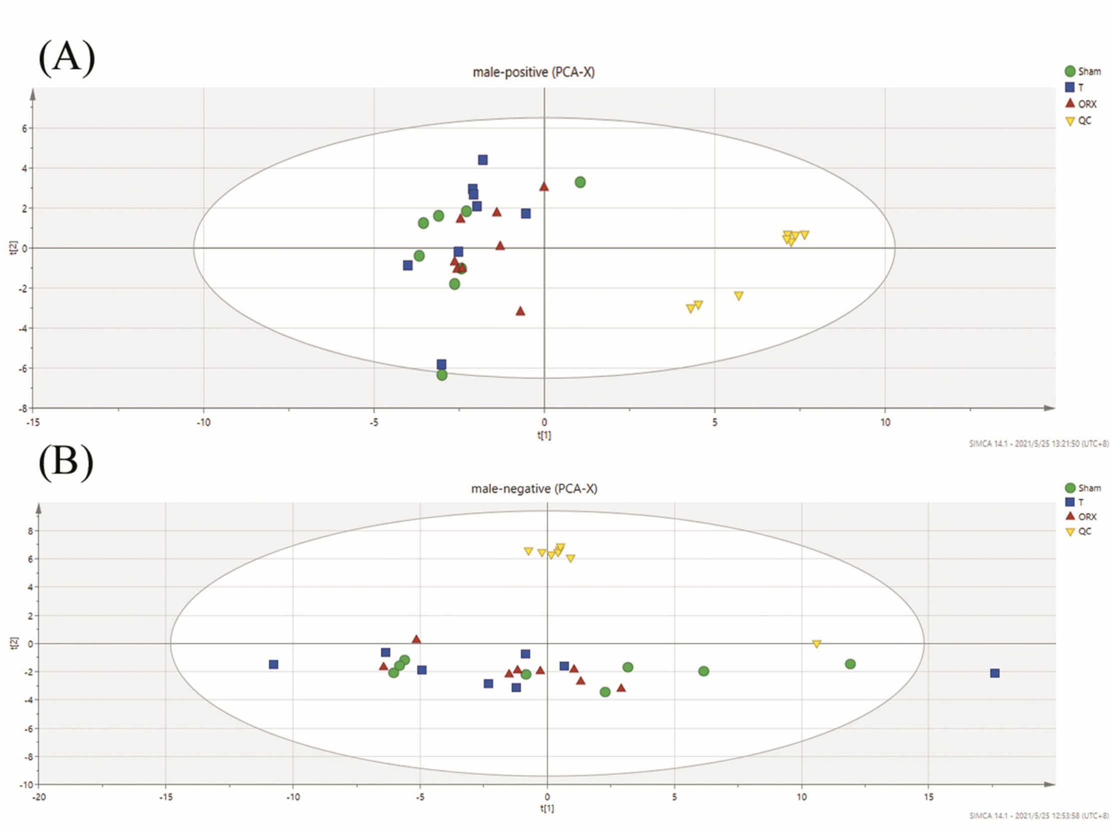

Figure S5. Score Scatter Plots for PCA-X analysis in (A) negative mode and (B) positive mode.

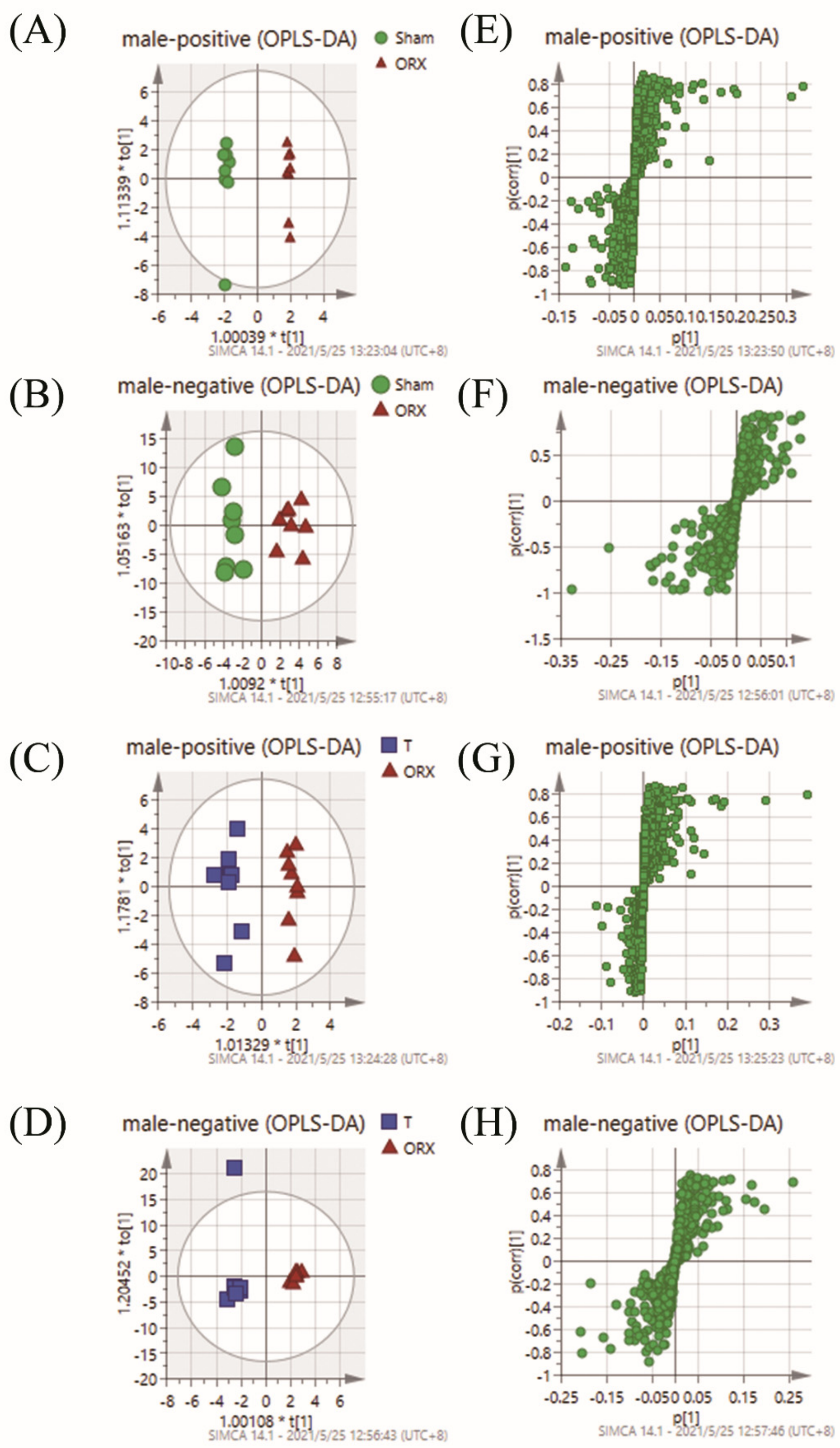

Figure S6. OPLS-DA score plots of Sham, ORX and T group (A–D) and their corresponding S-plot (E–H). Promising degree of fitting and predictive ability make it

reliable to screen the differential variables between groups: (A, E) ORX vs. Sham in positive mode; (B, F) ORX vs. Sham in negative mode; (C, G) ORX vs. T in positive mode; (D, H) ORX vs. T in negative mode.

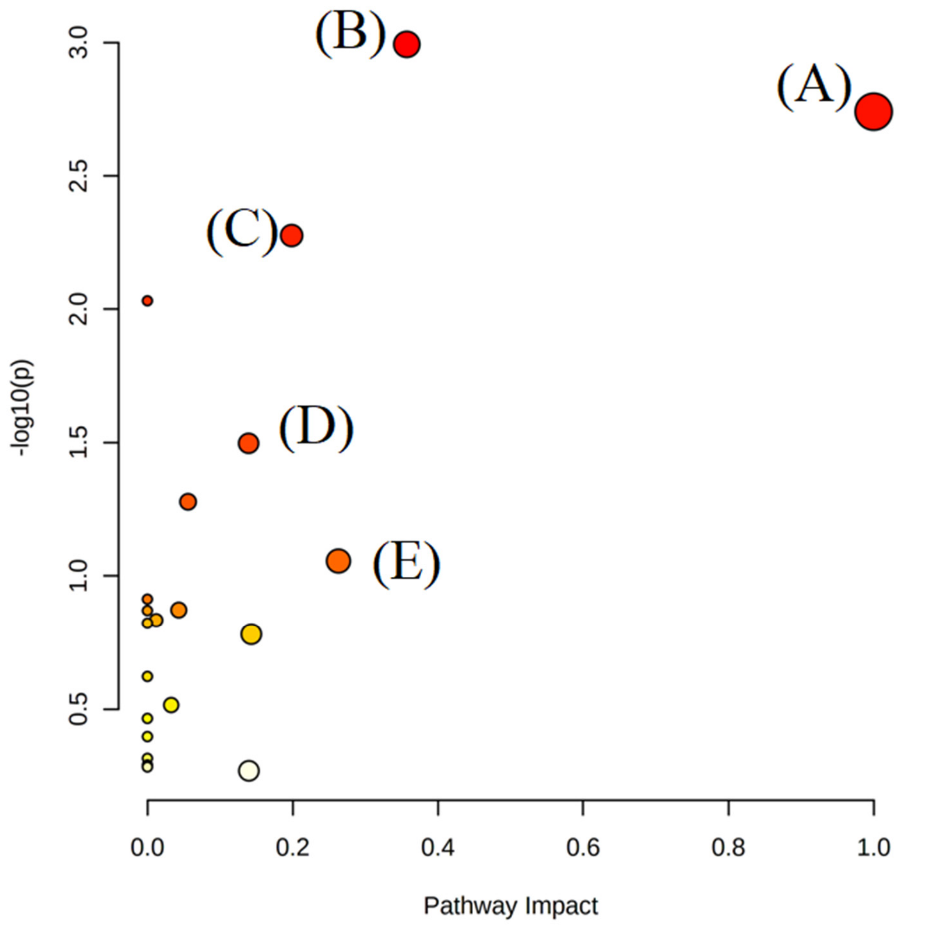

Figure S7. Pathway enrichment result of the differentiated metabolites (A) Phenylalanine, tyrosine, and tryptophan biosynthesis; (B) Phenylalanine metabolism; (C) Purine metabolism; (D) Histidine metabolism; (E) Glutathione metabolism.

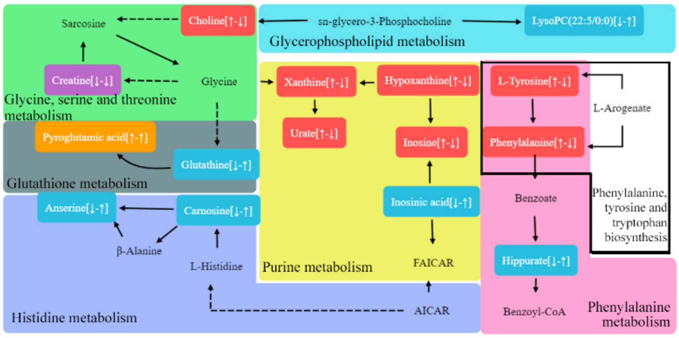

Figure S8. Metabolic pathways related to the differential metabolites identified in the Sham, ORX and T group.

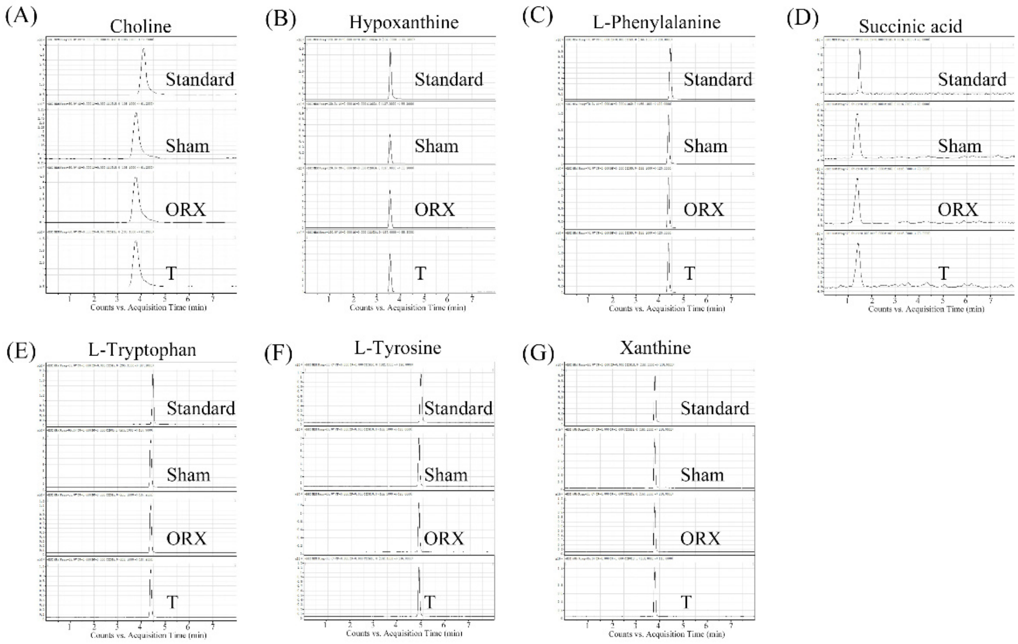

Figure S9. Extracted ion Chromatogram (EIC) from7 metabolites MRM analysis. A-G: EIC of choline, hypoxanthine, L-phenylalanine, succinic acid, L-tryptophan, L-tyrosine, xanthine in standard, sham, ORX, T group separately.
